# Direct and indirect cumulative effects of temperature, nutrients, and light on phytoplankton growth

**DOI:** 10.1002/ece3.70073

**Published:** 2024-07-31

**Authors:** Anna Lena Heinrichs, Onja Johannes Hardorp, Helmut Hillebrand, Toni Schott, Maren Striebel

**Affiliations:** ^1^ Institute for Chemistry and Biology of the Marine Environment (ICBM) Carl‐von‐Ossietzky University of Oldenburg, School of Mathematics and Science Oldenburg Germany; ^2^ Helmholtz Institute for Functional Marine Biodiversity (HIFMB) Carl‐von‐Ossietzky University of Oldenburg Oldenburg Germany; ^3^ Alfred Wegener Institute, Helmholtz‐Centre for Polar and Marine Research [AWI] Bremerhaven Germany

**Keywords:** cell size, cell stoichiometry, direct effects, gradient design, growth, indirect effects

## Abstract

Temperature and resource availability are pivotal factors influencing phytoplankton community structures. Numerous prior studies demonstrated their significant influence on phytoplankton stoichiometry, cell size, and growth rates. The growth rate, serving as a reflection of an organism's success within its environment, is linked to stoichiometry and cell size. Consequently, alterations in abiotic conditions affecting cell size or stoichiometry also exert indirect effects on growth. However, such results have their limitations, as most studies used a limited number of factors and factor levels which gives us limited insights into how phytoplankton respond to environmental conditions, directly and indirectly. Here, we tested for the generality of patterns found in other studies, using a combined multiple‐factor gradient design and two single species with different size characteristics. We used a structural equation model (SEM) that allowed us to investigate the direct cumulative effects of temperature and resource availability (i.e., light, N and P) on phytoplankton growth, as well as their indirect effects on growth through changes in cell size and cell stoichiometry. Our results mostly support the results reported in previous research thus some effects can be identified as dominant effects. We identified rising temperature as the dominant driver for cell size reduction and increase in growth, and nutrient availability (i.e., N and P) as dominant factor for changes in cellular stoichiometry. However, indirect effects of temperature and resources (i.e., light and nutrients) on species' growth rates through cell size and cell stoichiometry differed across the two species suggesting different strategies to acclimate to its environment.

## Graphical abstract

We evaluated the linear effects of temperature and the resources light and nutrients on the cell size, cell stoichiometry, and growth rate of two freshwater phytoplankton species, differing in their cell size. We used a structural equation model that identified the indirect and direct effects of the abiotic factors on the species' growth rates. We found different indirect effects of abiotic factors across species through cell size and stoichiometry changes, suggesting that the different species used different acclimation strategies.

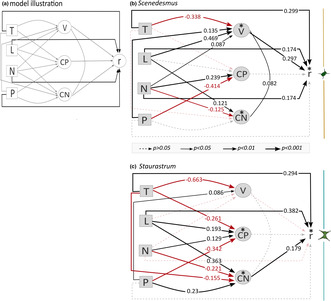

## 1 INTRODUCTION

Organisms are permanently facing changes in environmental conditions, but rate and amplitude of change in aquatic systems increased as a consequence of climate change and human activities associated with higher water temperatures, increased organic matter runoff, and altered nutrient loading (IPCC, 2023). For phytoplankton as primary producer, temperature and resource availability, such as light and nutrients, are among the strongest drivers determining population dynamics and community composition.

To understand and predict community dynamics under different environmental scenarios, an often used parameter is the growth rate of a population or whole community as it provides information on how phytoplankton performs in its environment. While many experimental studies tested for the effects of light, nitrogen, phosphorus, or temperature on phytoplankton growth, most of them focus on one of these factors keeping other factors at optimal conditions. Since multiple studies reported bivariate interactive effects between, for example, resources and temperature (e.g., Aranguren‐Gassis & Litchman, 2020; Boumnich et al., 1990; Hammer et al., 2002), the predictive power of studies focusing on solely one factor is questioned.

**FIGURE 1:**
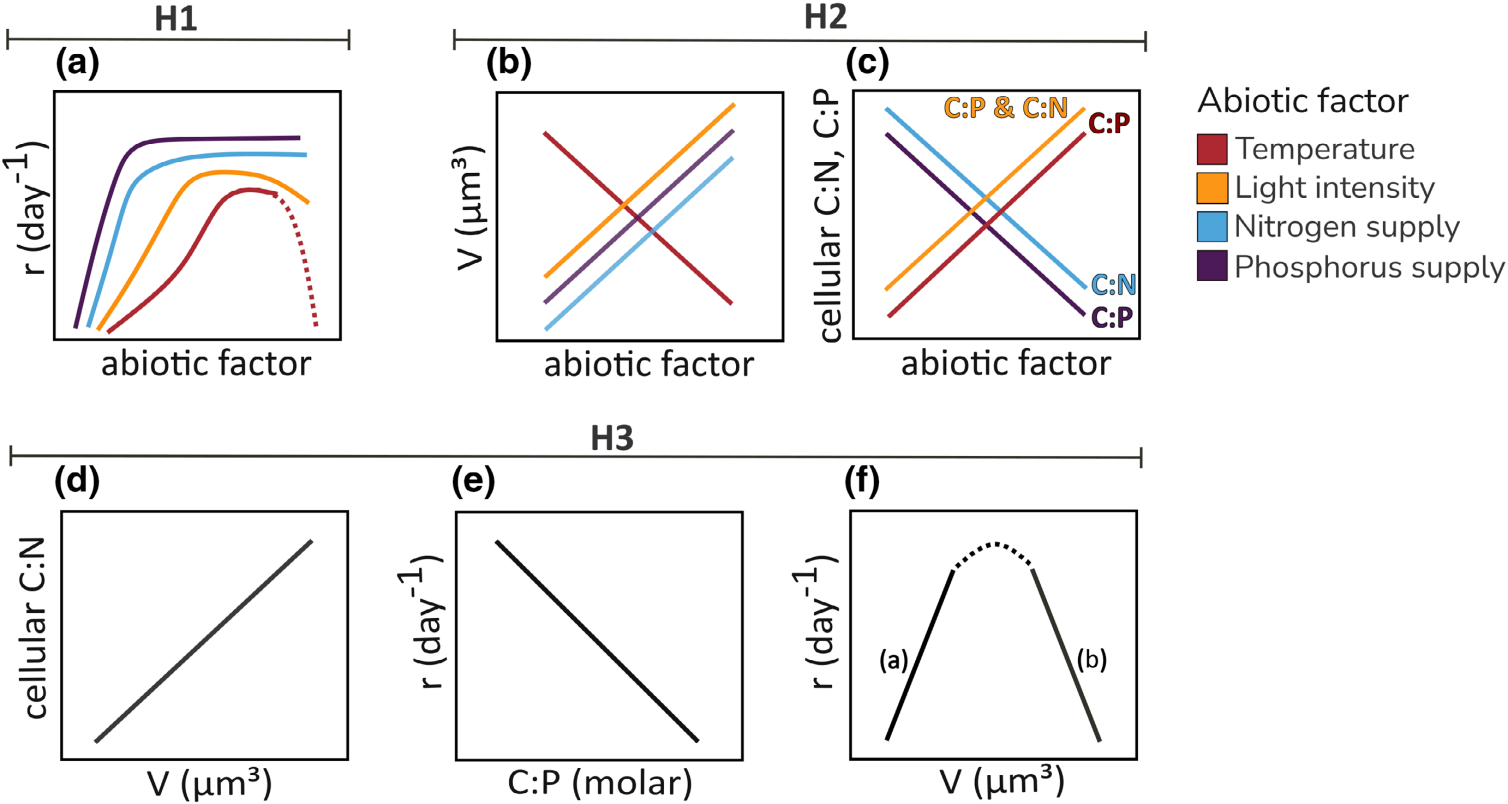
Assumed relationships between abiotic factors, growth rate, cell stoichiometry, and cell size for the species *Staurastrum* and *Scenedesmus*. Solid linear lines present the hypothesized effects tested in this study via SEM (H1‐H3). Dotted lines show the expected trends along a wider range of the abiotic factors demonstrated by other studies, but that are not expected to be found in this study (a) Assumed positive effects of temperature, light intensity, nitrogen, and phosphorus supply on the species‐specific growth rate r (H1). (b) Hypothesized effects of temperature, light intensity, nitrogen, and phosphorus on species' cell size V. (c) Hypothesized effects of temperature, light intensity, nitrogen, and phosphorus on either cellular C:P or C:N ratios or both. (d) Hypothesized positive correlation between cell size and cellular C:N ratio. (e) Hypothesized negative relationship between species' growth rates and C:P ratio. (f) Assumed relationship between species' growth rates and cell size. Solid lines show the linear relationship hypothesized in this study that differs between the two species: we hypothesized growth rate and cell size being positively related in the small species *Scenedesmus* (a) but negatively related in the large species *Staurastrum* (b) due to the unimodal trend found in other studies over a wider range of size classes (indicated by the dotted line).

Studies that involve multiple factors often use a binary approach, combining high and low levels of each treatment in a factorial manner. However, such binary options provide limited predictive power for models and transfer to nature, as they do not allow identifying response surfaces across the different, potentially interactive dimensions of environmental change (Thomas & Ranjan, 2023). To fill this gap, we conducted a multiple‐factor gradient experiment that tests the cumulative responses to the factors temperature, light, nitrogen, and phosphorus in phytoplankton growth. As the effects of temperature and resources on growth rate are, besides their direct effects, potentially driven by changes in cell size and elemental stoichiometry, we explicitly test for indirect effects of these factors on growth through changes in cell size and stoichiometry. Although a multitude of experimental designs across broad ranges of phytoplankton species exist reporting the effects of each factor on the growth rate, cell size, and cellular stoichiometry of phytoplankton, a fully mechanistic understanding of how resources and temperature affect the growth rate (H1), cell size and stoichiometry (H2), as well as their interdependencies (H3) at species level is less explored. We used a structural equation model (SEM) to test their effects on species‐specific growth rates, cell size, and stoichiometry, using the species *Scenedesmus armatus* and *Staurastrum manfeldtii*. These species were chosen based on their different cell sizes and growth characteristics to test the generality of the predicted effects. We formulated the following hypotheses for this experiment based on experiments testing for the individual effects of temperature, light, and nutrients on phytoplankton growth, cell size, and stoichiometry (Figure 1), and aim to provide a better understanding of how species performance is directly and indirectly linked to their environment, helping to predict community structure outcomes.

### 1.1 Direct effects of temperature and resources on growth rate (H1)

Generally, the growth response of a population is an unimodal left‐skewed function of temperature, where the growth rate increases with increasing temperature until the species‐specific temperature optimum is reached and growth declines sharply (Eppley, 1972; Montagnes et al., 2003; Thomas et al., 2012). With increasing light intensity, the growth rate also increases until a species‐specific optimum light intensity is reached, whereas higher light intensities can lead to reduced growth due to photoinhibition (Dauta et al., 1990; Edwards et al., 2015; Falkowski et al., 1985). Regarding the effect of nutrient concentrations, such as nitrogen and phosphorus, the growth rate increases with nutrient supply in a decelerating manner until it saturates at r_max_ at a species‐specific nutrient concentration (Eppley & Thomas, 1969; Qu et al., 2018). Based on that we hypothesize (H1): along the gradients of temperature and resource supply, growth rates are positively affected by light intensity, nitrogen (N), and phosphorus (P) supply, until the resources become saturating (Figure 1a). We also assume a positive temperature effect on growth. Thereby, we expect that the temperature range used will not lead to supra‐optimal conditions, at which growth would decline again and form an unimodal response, as a previous experiment with these species has shown increasing growth rates of up to 30°C (A. Heinrichs, A. Happe, H. Hillebrand, A. M. Koussoroplis, J. Merder, M. Striebel, unpublished) (Figure 1a).

### 1.2 Direct effects of temperature and resources on cell size and stoichiometry (H2)

Cell size is a master trait that is coupled with resource uptake and utilization strategies (Hillebrand, Acevedo‐Trejos, et al., 2022; Litchman & Klausmeier, 2008), and is therefore influenced by the availability of resources. Consequently, higher light intensity and nutrient concentration increase phytoplankton cell size at both, individual species level (Falkowski & Laroche, 1991; Hessen et al., 2002; Thompson et al., 1991) and community mean cell size level (Hillebrand, Di Carvalho, et al., 2022 for phosphorus effect, Peter & Sommer, 2013). Moreover, much attention has been given to the temperature dependence of phytoplankton cell size (Zohary et al., 2021) as cell size reduction is proposed to be the third universal response to climate warming (Daufresne et al., 2009) and thus directly linked to shifts in consumer size structure (Sommer et al., 2017; Venkataramana et al., 2019). In consideration of these findings, we hypothesize, regardless of the different levels of the other factors and their potential interactive effects, an increase in cell size with increasing light intensity and nutrient supply (N and P), but a reduction with rising temperature (H2a, Figure 1b).

Phytoplankton stoichiometry is highly flexible (Garcia et al., 2018) as it is driven by photosynthesis (C‐fixation) on the one hand and the uptake of nutrients (e.g., N and P) on the other hand. Consequently, the elemental stoichiometry of phytoplankton is primarily influenced by the availability of light and nutrients (Sterner et al., 1997; Sterner & Elser, 2002). Therefore, we hypothesize that cellular C:N and C:P ratios both increase with increasing light intensity but decrease with increasing N and P supply until resource requirements are met (H2b, Figure 1c). In addition to light and nutrients, phytoplankton stoichiometry is also controlled by temperature. Under colder conditions, more P‐rich ribosomes are needed to compensate for reduced efficiency in protein synthesis (Toseland et al., 2013). Hence, phytoplankton living at lower temperatures are associated with higher P content and consequently exhibit lower C:P and N:P ratios compared to phytoplankton living at higher temperatures (Peter & Sommer, 2015; Schaum et al., 2018; Yvon‐Durocher et al., 2017). Cellular C:N ratios in contrast seem to be more independent of temperature (Cotner et al., 2006; Verbeek et al., 2018; Yvon‐Durocher et al., 2017). Consequently, we expect cellular C:P to be negatively affected by P supply and positively affected by temperature and, in case biomass accumulation is limited by N, also by N supply (H2c, Figure 1c).

### 1.3 Relationship between growth rate and cellular size or stoichiometry (H3)

Cell size and stoichiometry mirror how resources are required and metabolized, and thus are intricately linked to growth rate. We therefore expect certain patterns to emerge from their responses to resource availability and temperature, and from their physiological interdependence. Larger cells have higher carbon fixation rates, nutrient uptake rates, and higher storage capacity for N (Hillebrand, Acevedo‐Trejos, et al., 2022). Thereby, the carbon content increases proportionally more with size relative to the N content leading to a positive relationship between cell size and cellular C:N ratio (Hillebrand, Acevedo‐Trejos, et al., 2022; Mei et al., 2011), especially under non‐limiting resource conditions (Mei et al., 2011). Therefore, we expect cellular C:N ratio to be positively related to increasing cell size (H3a, Figure 1d).

There is evidence that higher growth rates require more investment in P‐rich ribosomes. Consequently, fast‐growing species contain higher concentrations of P‐rich rRNA resulting in a negative relationship between growth and cellular C:P and N:P ratio (growth rate hypothesis, GRH) (Elser et al., 2010; Goldman, 1986), which has also been reported for phytoplankton studies (Elser et al., 2000; Hillebrand et al., 2013). However, the application of this hypothesis to phytoplankton is controversial as it has been shown that the limiting nutrient can influence the relationship between growth and stoichiometry (Flynn et al., 2010; Isanta‐Navarro et al., 2022). Therefore, we test here the generality of this relationship in phytoplankton using various levels of resources and temperature. In the case that growth rate and cellular P content are positively related, leading to a negative relationship between growth and cellular C:P ratio, we interpret the GRH as supported by our data (H3a, Figure 1e).

Across a broader spectrum of size classes, including picoplankton, phytoplankton growth rate shows a unimodal relationship with cell size, implying an optimal cell size for maximum growth around 10^3^ μm^3^ (Maranon, 2015; Maranon et al., 2013; Ward et al., 2017). As a consequence, cell sizes below the optimum are considered to be positively related to growth, while cell sizes above the optimum are negatively related to growth. Based on this relationship, we expect different size–growth patterns between *Staurastrum* and *Scenedesmus*, as they differ strongly in size (H3b, Figure 1f): the growth rate of the large species *Staurastrum* (cell size of 1756 μm^3^) is expected to be negatively related to cell size as its size is above the size optimum (>10^3^ μm^3^), while the growth rate of the small species *Scenedesmus* is positively related to cell size as its size lays below the size optimum (cell size of 39 μm^3^, thus <10^3^ μm^3^) (H3b, Figure 1f).

## 2 METHODS

### 2.1 Species selection

We conducted a laboratory experiment using two phytoplankton species, *Scenedesmus armatus* and *Staurastrum manfeldtii*, isolated from the freshwater lake Grafschaftssee (Germany, 53°33,005″ N; 7°58,049″ E) in July 2020 and identified based on morphological characteristics. By using these two species with different traits, we are able to check for the generality of our findings. For instance, they exhibit significant differences in cell size, with *Staurastrum* measuring over 40 times larger (1756 ± 10.2 μm^3^) than *Scenedesmus* (39 ± 0.6 μm^3^), however, only the latter is able to form colonies. Species isolation was conducted using a micropipette (Andersen & Kawachi, 2005) under an inverted microscope (Leica®). Isolation steps were repeated until a monoclonal culture was obtained for each species (cultures were unialgal but not axenic). Prior to the start of the experiment, species were cultivated in 1/4 WC Medium (Guillard & Lorenzen, 1972) at 18°C and a light intensity of 70 μmol photons m^−2^ s^−1^ with a 12:12 light:dark regime.

### 2.2 Experimental design

A multiple‐factor gradient experiment was performed with five levels of temperatures, five light intensities, five nitrogen concentrations, and five phosphorus concentrations, for each species, resulting in a total of 1250 experimental units (Table 1). Growth rates, elemental composition (C:N:P), and cell size of the two species were determined as response variables to the experimental conditions. The experiment with *Scenedesmus* started in November 2020, and the one with *Staurastrum* in February 2021. The experiments were conducted in cell culture flasks (Sarstedt AG & Co. KG) using a total volume of 50 mL. The bottles were incubated in the indoor mesocosms at the ICBM Wilhelmshaven (Gall et al., 2017) to ensure full light and temperature control. To obtain five different temperature levels, all samples were incubated using floating plastic boxes on the water surface of the mesocosm providing the respective temperatures (Table 1). Achieved conditions were controlled with continuous data loggers (HOBO Pendant®, Onset). The light conditions (Table 1) were established using an LED light setup on top of each mesocosm and reducing light with four different grey filter foils (LEE Filters, Filter nos. 209, 210, 211, and 298) covering the floating plastic boxes. The gray light filter foils reduced the light quantity but retained the full light spectrum, thus only the light intensity but not the quality differed between the light treatments. For the nutrient gradient, nitrogen (N as NaNO_3_) and phosphorus (P as K_2_HPO_4_) were added at the beginning of the experiment as a single addition in 25 different ratios (Table 1). To avoid limitations by other elements, we added nutrients, except N and P, according to 1/4 WC growth medium (Guillard & Lorenzen, 1972). As the species originated from oligotrophic conditions, we kept the medium reduced instead of using a full WC medium to ensure more realistic nutrient conditions.

**TABLE 1:** Experimental treatments.

Temperature °C		Light μmol photons m^−2^ s^−1^			Nutrient supply μmol L^−1^					
					**P**					
	**×**		**×**	**N**
**×**	**0.09**	**0.94**	**1.70**	**2.20**	**2.95**
10		36			**1.81**	20	2	1.1	0.8	0.6
15		62			**13.16**	146	14	8	6	4
20		135			**26.27**	292	28	15	12	9
25		183			**34.28**	381	36	20	16	12
30		264			**46.49**	517	49	27	21	16

*Note*: All treatments (temperature (°C), light intensity (μmol photons m^−2^ s^−1^), and initial nutrient supply (μmol L^−1^)) were set up in a multiple‐factor gradient design (5 × 5 × 5 × 5) resulting in 625 treatments per species. As nutrient supply, we added N (vertical bold written concentrations) and P (horizontal bold written concentrations) in 25 combinations of N:P ratios.

### 2.3 Sampling

For sampling, the cell flasks were removed from the incubators for a maximum of 1 h every second day. The optical density (OD, absorbance at 440 nm) and the raw fluorescence (RFU, excitation = 395 nm; emission = 680 nm) were measured using a microplate reader (Synergy H1, BioTek instruments) to track the biomass development over time. Flasks were gently shaken before sampling and 0.5 mL subsamples were removed and measured using 48‐well microplates (SARSTEDT AG & Co.KG). Sampling was performed under a Clean Bench to ensure sterile conditions when flasks were opened for sampling. After sampling, cell flasks were returned and replaced randomly in their respective light treatment boxes in the incubators. Samples for cell size and elemental composition determination (C:N:P) were only taken at the end of the experiment in the stationary phase. The stationary phase was defined as the biomass did not increase for at least 6 following days (three samplings), thus samples were finished at different time points depending on when they reached the stationary phase, or samples were finished when no growth was observed after at least 2 weeks.

### 2.4 Sample analyses

#### 2.4.1 Growth rates

Growth rates (day^−1^) were determined as the exponential growth rate by selecting the exponential part of the curve (Hall et al., 2014) with the R package “*growthrates*” and the command *“fit_easylinear”* (Petzoldt, 2022) using the RFU data (Figure S1). This command fits segments of linear models to the log‐transformed data to find the maximum growth rate. RFU instead of OD data were used as they showed a stronger correlation with species' abundance based on microscopic cell counts (Figure S2). Note that the cultures were not acclimatized prior to the experiment, which could bias the growth rate estimates on the first days. Since we started with low cell densities that allowed a lag phase before growth started and ran the experiment until the populations reached their stationary phase (duration of at least 10 days), we are confident that acclimation did not alter the overall results of the growth rate estimations (see Figure S1 for growth curves).

#### 2.4.2 Cell size

To test how the cell size (in μm^3^) of the species changed under the experimental conditions, we fixed subsamples with Lugol's iodine solution at the end of the experiment (10 vol% final concentration), when the population had reached the stationary phase. For the small species *Scenedesmus*, we used the CoulterCounter (Beckman Z2 particle counter) for cell size determination, which determines cell size as equivalent sphere volume. As *Scenedesmus* tends to form either chains of four single cells or two single cells, which were identified as one large cell by the CoulterCounter (cell size in sphere volume), we tested for the cell size of these different colonies to distinguish between them (Figure S3 for cell counter distribution of single cells and colonies). To get the cell size of the single cells, cell volumes larger than 100 μm^3^ were identified as four chain colonies and divided by a factor of 4, and cell volumes smaller than 100 μm^3^ were identified as two chain colonies and divided by a factor of 2 (see Figure S3). For *Scenedesmus*, the sphere volume (in μm^3^) was calculated using the diameter of the cell measured by the CoulterCounter. For the larger species *Staurastrum*, cell size determination, in μm^3^, was done via microscope (Axiovert 10, Zeiss) as the species was too large for the Cell Counter capillary. Cell dimensions were determined with the Image software ImageJ, measuring cellular dimensions of at least 20 individuals per sample with the cell size calculation methods by Hillebrand et al. (1999).

#### 2.4.3 Stoichiometry

Samples for elemental composition were taken in the stationary phase. For measurements of particulate organic carbon (POC), nitrogen (PON), and phosphorus (POP), samples were filtered (each 10–15 mL sample volume) onto acid‐washed precombusted glass fibre filters (Whatman GF/C) and stored at −20°C until analysis. Filters for POC and PON were dried at 60°C for 4 days, put in tin capsules, and analyzed via an elemental analyzer (Flash EA 1112, Thermo Scientific). Phytoplankton filters for POP were combusted at 400°C and measured with molybdate reaction based on the method by Wetzel and Likens (2000) after digestion with potassium peroxydisulfate (K_2_S_2_O_8_) solution.

#### 2.4.4 Structural equation model

We performed the statistical analysis in R, version 3.6.2 (the R Foundation for Statistical Computing Platform).

To investigate the effects of the abiotic factors (temperature, light, N, and P) on phytoplankton growth, cell size, and stoichiometry, as well as to analyze the relationships between these response variables to identify indirect effects (Figure 1), we used a piecewise structural equation model (SEM) using the piecewise SEM package (Lefcheck, 2016). An SEM combines multiple linear relationships thus interacting processes between variables are considered, and shows the network of links between all variables, between both abiotic factors and response variables (H1 and H2), as well as between the response variables (H3).

We fitted the linear models used for the SEM based on the hypotheses we formulated (Figure 1). Since not all responses are linear, we tested for non‐linear effects by implementing quadratic terms in the linear model and selected the model with the best AIC (see Figures S8–S11 for model validation plots and more details on the SEM). Implementing non‐linear terms in the linear model improved in most cases the fit (Table S1) but did not change the direction of the effect compared to models including only linear effects. We used the four abiotic factors (temperature, light, N, and P) as exogenous variables and the four response variables (cell size, growth rate, and C:N and C:P ratio) as endogenous variables. Endogenous variables were tested for normal distribution and transformed when necessary before modeling (Figure S4). We scaled all exogenous variables in order to standardize the regression coefficients and allow for a comparison of effect sizes (Schielzeth, 2010). In addition to the effects of the exogenous variables, we also implemented one correlation term in the SEM between the endogenous variables C:N ratio and cell size based on hypothesis H3a (Figure 1d). For the SEM, it is necessary that the number of observations is equal for each tested variable. Therefore, we lost some data values due to excluded outliers of PON measurements and lost samples due to errors in the CN‐analyzing process, resulting in 512 observations in total instead of 625 for *Scenedesmus* and 444 observations in total for *Staurastrum*. It should be noted that our SEM comprised all potential paths of the treatment and measured variables, but this does not preclude that other unobserved mechanisms are relevant and would change the outcome of the model.

## 3 RESULTS

### 3.1 Direct effects of abiotic factors on growth (H1)

**FIGURE 2:**
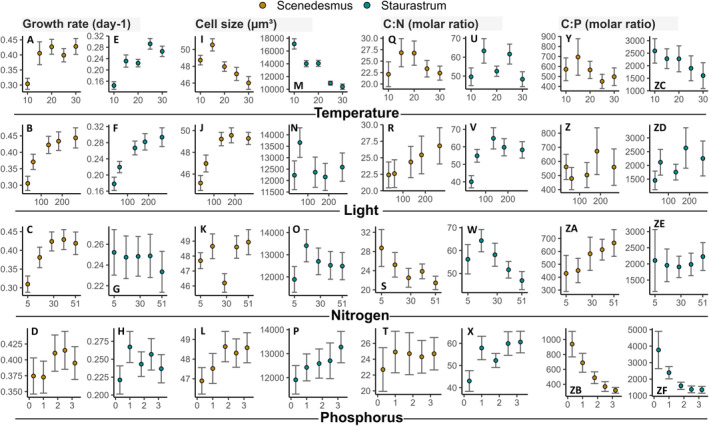
Observed data of the response variables growth rate (A‐H), cell size (I‐P), cellular C:N ratio (Q‐X), and C:P ratio (Y‐ZF) along the gradients of temperature (°C), light intensity (μmol photons m^−*2*^ s^−*1*^), nitrogen (μmol L^−*1*^), and phosphorus (μmol L^−*1*^). The colored circles present the mean values and the error bars its standard errors for each treatment level (the average response contains all other treatment levels, thus *n* = 125). For inspection of the absolute elemental content (carbon, nitrogen, and phosphorus), see Figure S5.

**FIGURE 3:**
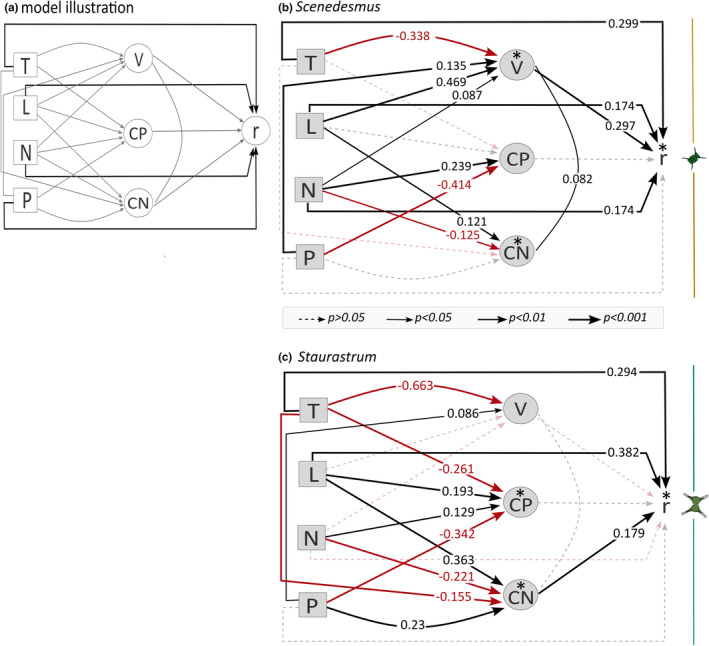
Structural equation model (SEM) to test (i) the effects of temperature, light, nitrogen, and phosphorus in a gradient design on species‐specific growth rates (r), cell size (V), and elemental composition (C:N and C:P ratios); and (ii) the relationships between species‐specific growth rates and cell size and stoichiometry. Values give the standardized slope estimates of the linear models. Red lines and values present negative slopes (thus, negative relationships), and black lines and values positive slopes (thus, positive relationships). Thickness of lines shows the significance level of the relationship (see legend box). * Gives the responses where non‐linear terms of the variables were included for the SEM (see Tables S1 and S2). Transparent dashed lines present relationships where the slope did not differ significantly from 0. Arrows give causal pathways. Lines without arrows present relationships without a direction (correlation term). Some response variables were transformed for the SEM (see Section 2 and Figure 4). (a) Initial model structure used for both species according to the hypotheses. Black lines show direct effects on growth rate, and gray lines the effects on cell size and stoichiometry and the indirect effects on growth. (b) SEM results of the small species *Scenedesmus*. (c) SEM results of the large species *Staurastrum*.

In both species, growth rates increased with increasing temperature (Figure 2a,e) and light (Figure 2b,f), resulting in significant direct effects in the SEM (Figure 3). By contrast, direct nutrient effects on species' growth rates were less consistent between the two species, as the SEM only revealed increasing growth with increasing N for *Scenedesmus* (Figure 2c,g and Figure 3). Growth was not directly affected by P supply in any of the two species (Figure 2d,h and Figure 3). The path coefficients for the direct effects ranged from 0.174 to 0.382 and thus were in a comparable range (Figure 3).

### 3.2 Treatment effects on cell size and stoichiometry (H2)

Cell size declined with increasing temperature in both species (Figure 2i,m and Figure 3). This effect was much stronger for the larger species *Staurastrum* (Figure 3). While cell size was positively affected by P supply in both species (Figure 2l,p and Figure 3), light intensity and N supply also positively affected cell size of *Scenedesmus* but not of *Staurastrum* (Figure 2j,k,n,o, and Figure 3).

In both species, cellular C:N ratio increased with increasing light intensity but decreased with N supply (Figure 2r,s,v,w, and Figure 3). In the large species *Staurastrum*, C:N ratio increased also with P supply and decreased with temperature (Figure 2u,x and Figure 3). Cellular C:P ratio increased with N supply but decreased with P supply in both species (Figure 2za,zb,ze,zf, and Figure 3), while the C:P ratio of *Staurastrum* increased with increasing light intensity but decreased with rising temperature (Figure 2zc,zd and Figure 3).

### 3.3 Relationship between growth rate and cellular size or stoichiometry (H3)

**FIGURE 4:**
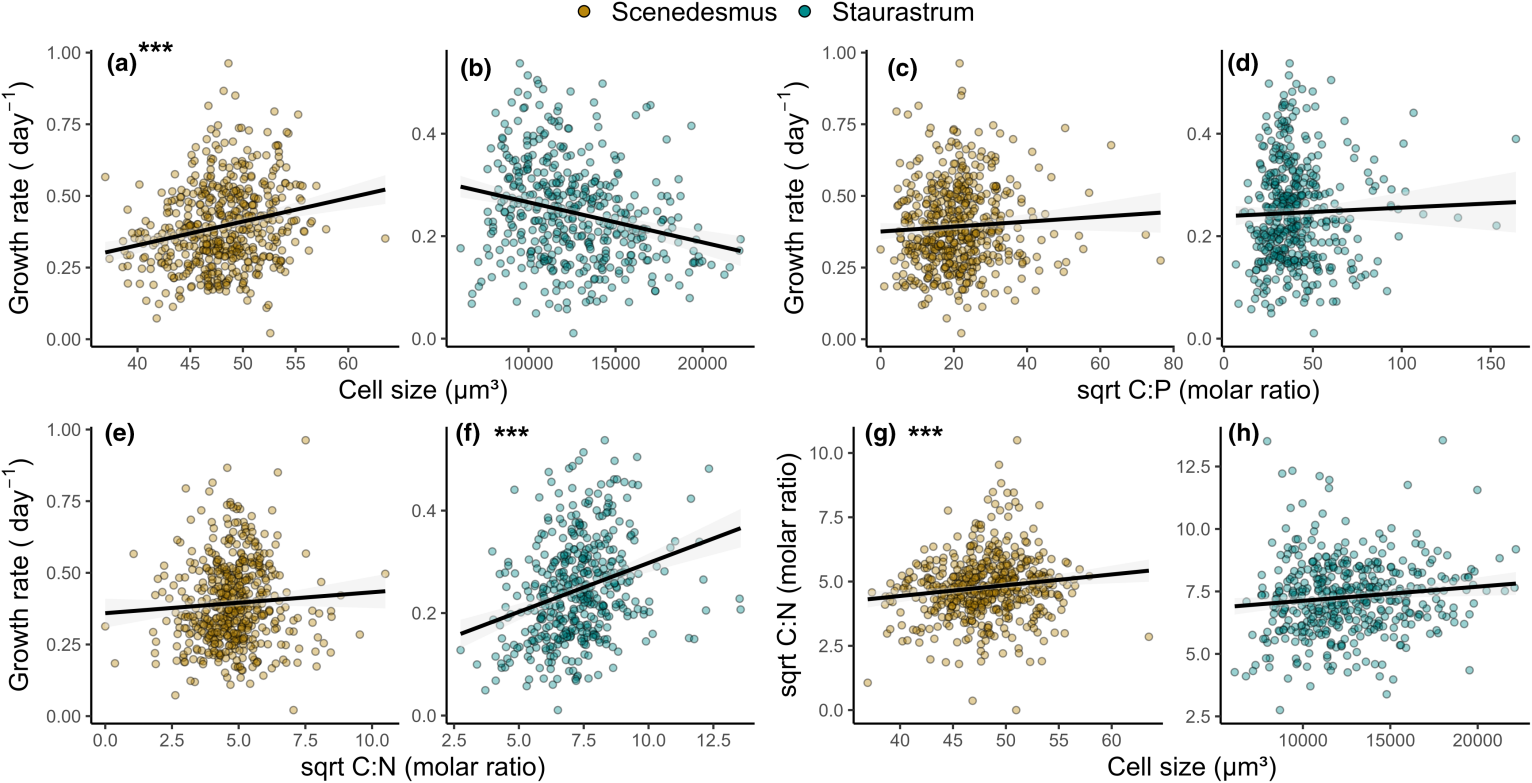
Relationship among species‐specific growth rates, cell size, and cellular stoichiometry using data for the SEM.Relationship between growth and cell size for *Scenedesmus* (a) and *Staurastrum* (b). Relationship between growth and cellular C:P ratio for *Scenedesmus* (c) and S*taurastrum* (d). Relationship between growth and cellular C:N ratio for *Scenedesmus* (e) and *Staurastrum* (f). Relationship between cellular C:N ratio and cell size for *Scenedesmus* (g) and *Staurastrum* (f). Solid black lines represent linear regressions for data that were used for the SEM. Stars in the upper left corner (***) mark relationships that were detected as significant by the SEM (*p* value<.05). Note here that some variables (C:N and C:P) are transformed as used for the SEM for normal distribution.

For the smaller species, *Scenedesmus*, there was only a significant positive link between cell size and growth (Figure 3 and Figure 4a). As cell size increased with increasing resource supply in this species, this can be seen as strengthening the direct light and N effect on growth and establishing an indirect link between P supply and growth. Conversely, temperature had positive effects on growth, but negative on cell size, resulting in a negative indirect temperature effect on growth potentially weakening the positive direct temperature effect in *Scenedesmus*. For the larger species *Staurastrum*, cellular C:N ratio and growth were positively related (Figures 3c and 4f), which can be seen as strengthening the direct temperature, light, and N effect on growth and establishing an indirect link between P supply and growth. Furthermore, cell size and cellular C:N ratio, which both increased with increasing light intensity, were positively related in *Scenedesmus* (Figure 3b and Figure 4g) but not in *Staurastrum* (Figure 3c and Figure 4h).

## 4 DISCUSSION

### 4.1 Direct effects of abiotic factors on growth rate (H1)

Within the range of temperature and resources we used, species‐specific growth rates were expected to increase with increasing temperature and resource availability (H1). For both species, temperature and light increased species‐specific growth rates, as hypothesized, thus dominating potential interactive effects between some of these factors which could buffer the main effects (Bestion et al., 2018; Bouterfas et al., 2002; Thomas et al., 2017). However, nutrient effects on growth rates differed between the two species and between nutrient types (i.e., N and P), indicating species‐specific resource demands. The differences in resource limitation between the two species are not surprising, considering that resource utilization, such as the photosynthetic response to light or nutrient uptake, is size dependent (Hillebrand, Acevedo‐Trejos, et al., 2022; Malerba et al., 2017; Ward et al., 2017). Large species are able to store more nutrients to overcome unfavourable conditions (Hillebrand, Acevedo‐Trejos, et al., 2022), and tend to exhibit a lower slope of the growth–irradiance curve (Edwards et al., 2015). This in turn suggests that larger phytoplankton tend to perform poorly under low light, while the storage of nutrients allows them to cope with nutrient‐poor conditions. This could explain why the larger species *Staurastrum* showed only a direct influence of light and not of N or P supply. In addition, P supply also had no direct effect on the growth of *Scenedesmus*, which corresponds to the fact that green algae have higher optimal cellular N:P ratios compared to other groups (Hillebrand et al., 2013), indicating that they are more likely to be limited by N rather than by P. Furthermore, the non‐significant nutrient effect on growth could indicate an interactive effect between nutrients and the other factors, as reported in several other studies (Choi et al., 2022, A. Heinrichs, A. Happe, H. Hillebrand, A. M. Koussoroplis, J. Merder, M. Striebel, unpublished; Thomas et al., 2017) which can change the direction of the response depending on the other factor level, thereby buffering the main effect.

### 4.2 Treatment effects on cell size and stoichiometry (H2)

As hypothesized, we observed a reduction in cell size with rising temperature, which aligns with numerous prior studies demonstrating a decline in cell size under elevated temperatures in phytoplankton communities (e.g., Hillebrand, Di Carvalho, et al., 2022; Peter & Sommer, 2013; Yvon‐Durocher et al., 2011; Zohary et al., 2021) and single species (Bernhardt et al., 2018; Hofmann et al., 2019). Our data thus support the suggestion made by Daufresne et al. (2009) that a reduction in body size represents the “third universal ecological response to global warming.” The enhanced temperature sensitivity observed in the cell size of *Staurastrum*, in contrast to *Scenedesmus*, is consistent with previous research indicating that temperature‐induced changes in cell size are more pronounced in larger phytoplankton than in smaller phytoplankton (Peter & Sommer, 2012). Moreover, both *Scenedesmus* and *Staurastrum* increased in size with rising P supply, but not with N supply. This finding is in line with an increase in the community‐weighted mean cell size of Wadden Sea phytoplankton associated with increasing P concentration but not with increasing N concentration (Hillebrand, Di Carvalho, et al., 2022).

Cellular C:P ratio of *Staurastrum* decreased with rising temperature, and for *Scenedesmus*, the relationship between temperature and cellular C:P, although not significant, tended to be negative as well. These results contradict previous studies that demonstrated elevated cellular C:P ratios in warmer temperatures (Peter & Sommer, 2013; Schaum et al., 2018; Verbeek et al., 2018; Yvon‐Durocher et al., 2017). Moreover, these findings appear to deviate from the *temperature‐dependent physiology* hypothesis, which posits a reduction in the content of phosphate‐rich ribosomes at higher temperatures, leading to elevated cellular C:P and N:P ratios (Elser et al., 2010; Toseland et al., 2013). The results found here can be neither explained by the *growth rate hypothesis* (GRH), which posits that P content and growth rate are positively related due to a higher demand of phosphate‐rich ribosomes for higher growth, resulting in a negative relationship between cellular C:P ratio and growth (Elser, Acharya, et al., 2003; Elser, Kyle, et al., 2003; Goldman et al., 1979; Isanta‐Navarro et al., 2022). Here, growth rates and cellular P content were not positively related, as cellular P content did not increase notably with rising temperature, as growth did (see Figure S5 for absolute cellular P content). Therefore, the negative effect of temperature on cellular C:P ratio found for *Staurastrum* does not align with the assumed patterns derived from the literature. The stoichiometric response to temperature has shown to be influenced by the availability of nutrients as it has been demonstrated in the study by Verbeek et al. (2018). They manipulated nutrients and temperature and found a significantly higher C:P ratio in the warmer treatment only under oligotrophic conditions. Here, we pooled multiple nutrient levels for each temperature, thus potential interactions could buffer the expected positive temperature effect on C:P ratios, which could be attributed to a lack of sufficient nutrient limitation in *Staurastrum*.

Nevertheless, the increase in C:nutrient ratio with increasing light, and the decrease in C:nutrient ratio with increasing nutrient supply found in this study is in accordance with the light:nutrient hypothesis that proposed a dependency of cellular C:nutrient ratio on the supplied light:nutrient ratio (Elser, Acharya, et al., 2003; Elser, Kyle, et al., 2003; Sterner et al., 1997). In conclusion, we found all hypothesized effects of resources on cellular stoichiometry in *Staurastrum* and almost all in *Scenedesmus* which support the generality of the light:nutrient hypothesis. Different from most studies, in this approach, we pooled multiple levels of multiple other factors which allowed us to illustrate that the resource‐driven effects on stoichiometry appear to predominate, even in the presence of potential interactions documented in prior studies. Furthermore, cellular C:P ratio increased with N supply in both species due to increases in cellular carbon content with N supply. The cross‐effects of P on N quota and N on P quota have been quite generally studied (Bracken et al., 2015) and are often signatures of co‐limitation. N limitation can reduce chlorophyll content and thereby limit carbon fixation, which would explain the increase in C:P when N was added.

### 4.3 Relationship between growth rates and cellular size or stoichiometry (H3)

We linked cell size and stoichiometry to species‐specific growth rates and tested for the generality of the relationships found in other studies, under the influence of different abiotic conditions. The observed relationships were only partly in accordance with the hypotheses we formulated and suggest a context dependence. Prior research has already demonstrated that the relationships between phytoplankton growth rate and cell size or stoichiometry are not necessarily strict but depend on the environmental conditions. Here, the hypothesized negative relationship between growth rate and stoichiometry (cellular C:P ratio), as predicted by the *growth rate hypothesis* (GRH), was not evident. Previous studies have reported N:P growth relationships that do not conform strictly to the GRH due to their dependence on the limiting nutrient (Flynn et al., 2010; Garcia et al., 2018; Hillebrand et al., 2013). A meta‐analysis conducted by Hillebrand et al. (2013) revealed a general negative relationship between growth rate and cellular N:P ratio across aquatic systems and taxa, but the decline was mainly induced by experiments under P limitation. In our study, both species were more constrained by N than by P. Based on findings by Hillebrand et al. (2013), this could explain why we did not observe the hypothesized negative relationship between growth rate and the C:P ratio. Although a link between cellular C:P ratio and growth was not evident, we found a link between cellular C:N ratio and growth in *Staurastrum*, suggesting that indirect effects of abiotic factors on growth via stoichiometry, modulating the relationship between growth and stoichiometry, occurred at least partly in *Staurastrum* but not in *Scenedesmus*.

The hypothesized relationship between growth and cell size, which was assumed to differ between species (positive for *Scenedesmus* and negative for *Staurastrum*) was only detected in *Scenedesmus*. Although there was a negative trend in *Staurastrum*, this relationship between growth and cell size was not significant. In addition to the temperature‐induced reduction in size in both species, *Scenedesmus* enlarged its cell size with increasing resource availability, but not *Staurastrum*. This suggests that *Scenedesmus* showed a higher plastic response to changing resource conditions relative to *Staurastrum*, which potentially allowed *Scenedesmus* to exhibit higher growth rates. In contrast, the cell size of *Staurastrum* was predominantly controlled by temperature which could have decoupled the growth–size relationship in *Staurastrum*. This agrees with findings by Sal et al. (2015), who found the unimodal growth–size relationship to be weaker at higher temperatures when analyzing data from 194 species assembled by Thomas et al. (2012). Hence, how phytoplankton respond to its environment seems to influence the growth–size relationship, which agrees with other studies. For instance, Mei et al. (2011) demonstrated that the overall negative growth–size relationship became weaker when nutrients were high or light was low. Similarly, Berges and Harrison (1995) observed, at the species level, a negative growth–size relationship under nutrient‐limited conditions but a positive relationship under light‐limited conditions. Here, although the temperature effect on growth and cell size was the same for both species, cell size was differently affected by resources between species, ending in different growth–size relationships. As a consequence, the indirect effects of abiotic factors on growth via cell size, which modulated the relationship between growth and cell size, occurred in *Scenedesmus* but not in *Staurastrum*.

## 5 CONCLUSION

In this study, we tested for the cumulative effects of temperature and resources (i.e., light, N, and P) on phytoplankton growth, cell size, and stoichiometry, which allowed us to identify the mechanisms that enable species to acclimate to its environment. Even under multiple levels of light and nutrients, we found the direct effects of temperature on growth and cell size that were detected in many other studies, thus temperature can be identified as dominant driver for growth and cell size. The hypothesized nutrient effect on stoichiometry, demonstrated in other studies, could also be proven here in both species. However, temperature effect on stoichiometry and resource effect (i.e., light and nutrients) on cell size were not consistent across species. Furthermore, the relationships between growth and stoichiometry, as well as growth and cell size, varied among the species, suggesting that these species used different strategies to acclimate to its environment. These results give us powerful insights into how species' responses in stoichiometry and size to abiotic conditions shape the indirect effects on growth and which acclimation strategies they use.

## AUTHOR CONTRIBUTIONS

**Anna Lena Heinrichs:** Conceptualization (equal); data curation (lead); formal analysis (lead); investigation (lead); methodology (lead); visualization (lead); writing – original draft (lead); writing – review and editing (lead). **Onja Johannes Hardorp:** Data curation (supporting); formal analysis (supporting); resources (equal); validation (supporting); writing – review and editing (supporting). **Helmut Hillebrand:** Conceptualization (equal); funding acquisition (lead); methodology (supporting); supervision (supporting); writing – review and editing (supporting). **Toni Schott:** Data curation (supporting); formal analysis (supporting); writing – review and editing (supporting). **Maren Striebel:** Conceptualization (equal); funding acquisition (lead); project administration (lead); supervision (lead); writing – original draft (supporting); writing – review and editing (supporting).

## CONFLICT OF INTEREST STATEMENT

We declare that there are no conflicts of interests.

## Supporting information


Data S1.

## Data Availability

The data and R codes used to generate the results in this paper are available in the public repository Zenodo (https://zenodo.org/records/12731714).
