# Supplementary material for: Direct and indirect cumulative effects of temperature, nutrients, and light on phytoplankton growth
Source: Ecol Evol. 2024 Jul 31;14(8):e70073. doi: 10.1002/ece3.70073 (PMC11289788; doi:10.1002/ece3.70073)
Supplement: Supplementary file 1 — Data S1. [file ECE3-14-e70073-s001.pdf]

Appendix: Anna Lena Heinrichs, Onja Johannes Hardorp, Helmut Hillebrand, Toni Schott and Maren Striebel. Direct and indirect cumulative effects of temperature, nutrients and light on phytoplankton growth.

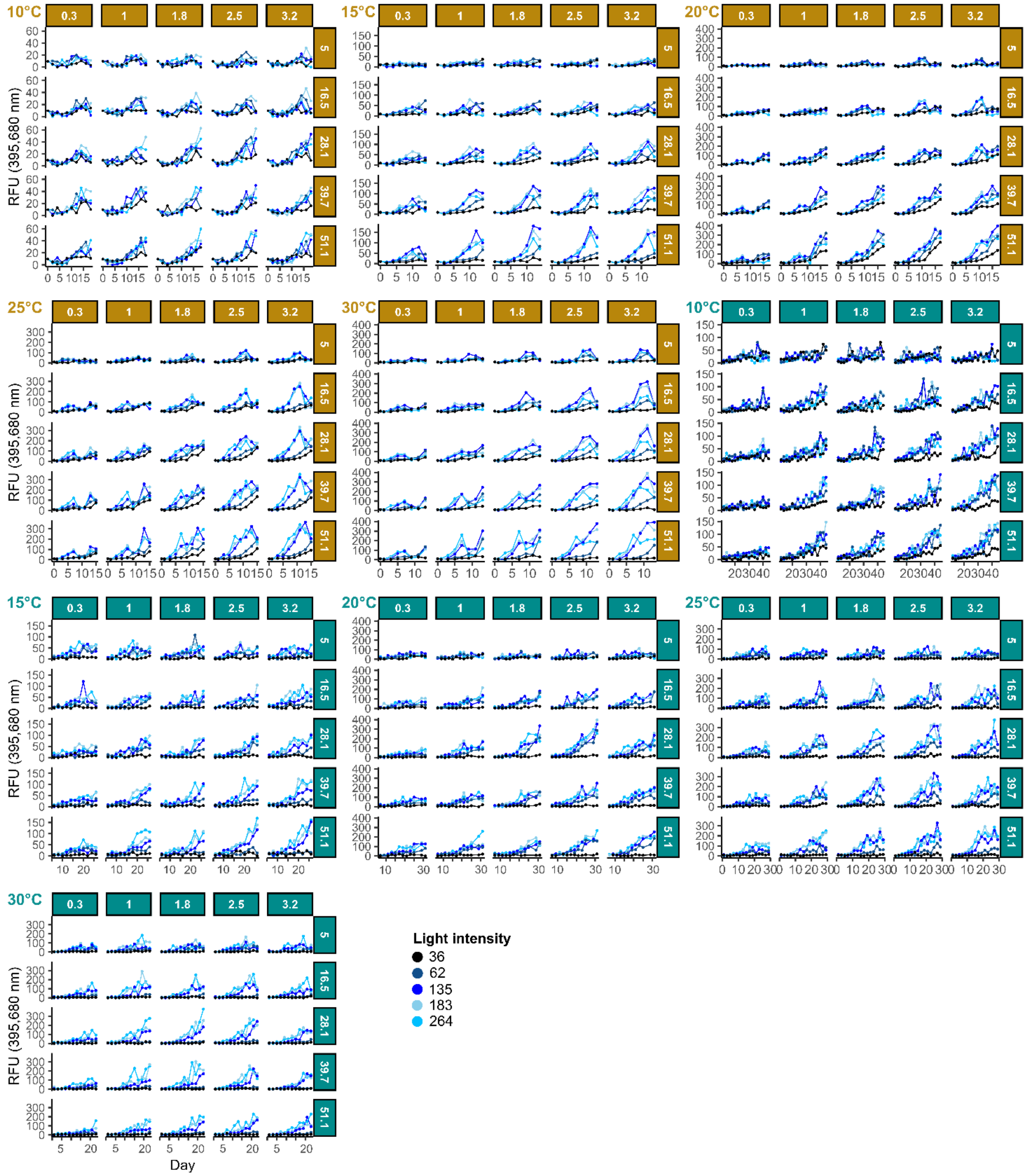

Figure S 1 Incubation data used for growth rate calculations, per species and treatment. Panel grids represent the initially supplied nitrogen (vertical direction) and phosphorus (horizontal direction) concentrations and coloured lines and circles the light intensity. Plots with gold-colored strips present data from *Scenedesmus*, and turquoise strips present data from *Staurastrum*.

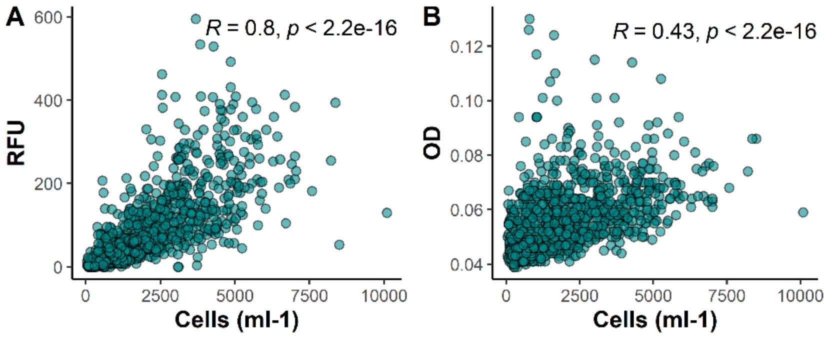

Figure S 2: Comparison between cell counts (determined microscopically) and Raw Fluorescence (RFU, **A**) and Optical Density (OD, **B**) in the species *Staurastrum*. The RFU measurements during the experiment were better correlated to cell counts compared to the OD measurements (indicated by a higher Pearson correlation coefficient *R*). For this reason, we used the RFU data as a biomass proxy for growth rate calculations.

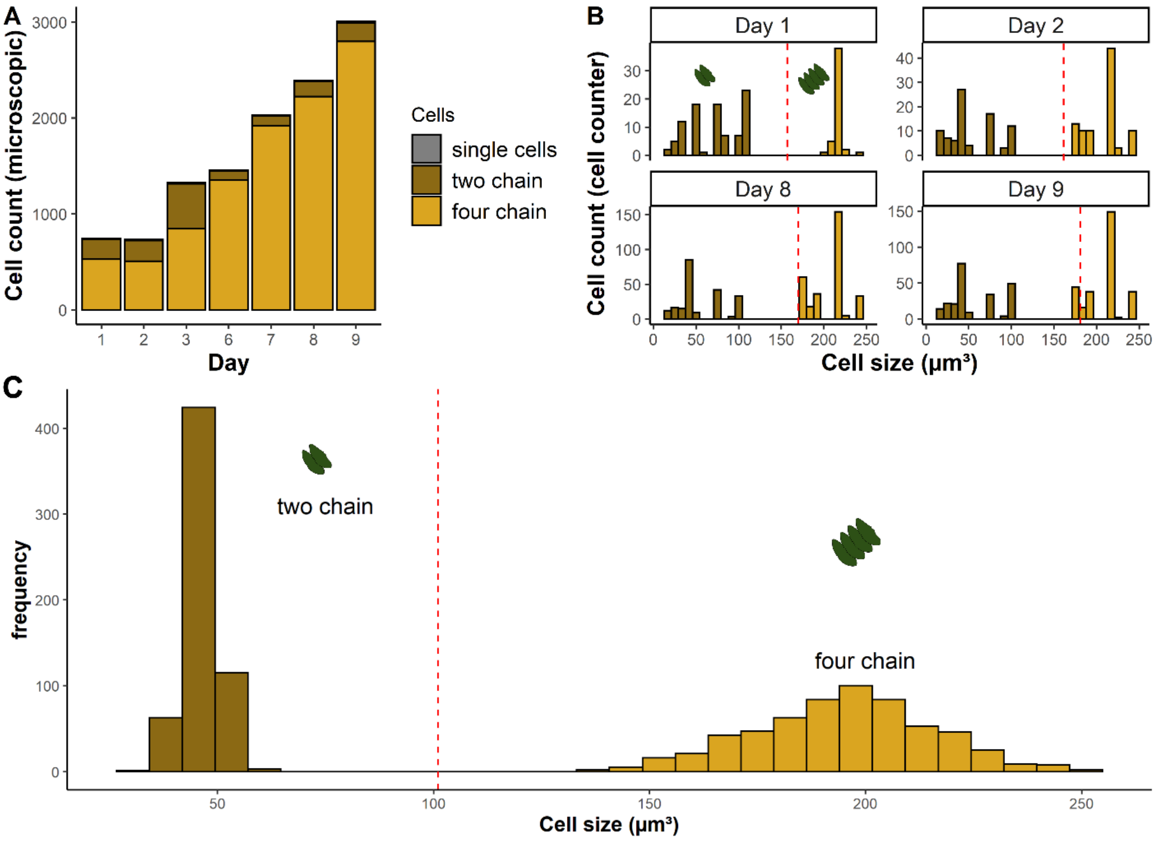

Figure S 3 Coulter Counter distribution for two chain cells and four chain cells in *Scenedesmus*. Output of the Coulter Counter measurements showed two peaks for cell size analysis which was due to the ability of *Scenedesmus* to form colonies. To avoid the colonies being counted as one large cell (which would lead to misestimating the cell size), we distinguish between these colonies by testing microscopically which colonies cause the different peaks. **A–B** We incubated *Scenedesmus* for 9 days and took subsamples to determine **A** The number of the different colonies microscopically and **B** to compare the size distribution given by the Coulter Counter with the microscope data. The amount of four chain cells (golden bars), which dominated in the sample, increased over time (A) and with that the mean cell size (B, red dashed line). The other peak (dark golden bars) presents two chain cells. **C** Therefore, for the experimental data, we split the Coulter counter data into two size classes. We identified cell sizes that were larger than 100µm³ as four chain colonies and divided the cell size by a factor of 4. Cell sizes below 100µm³ were identified as two chain cells and divided by a factor of 2.

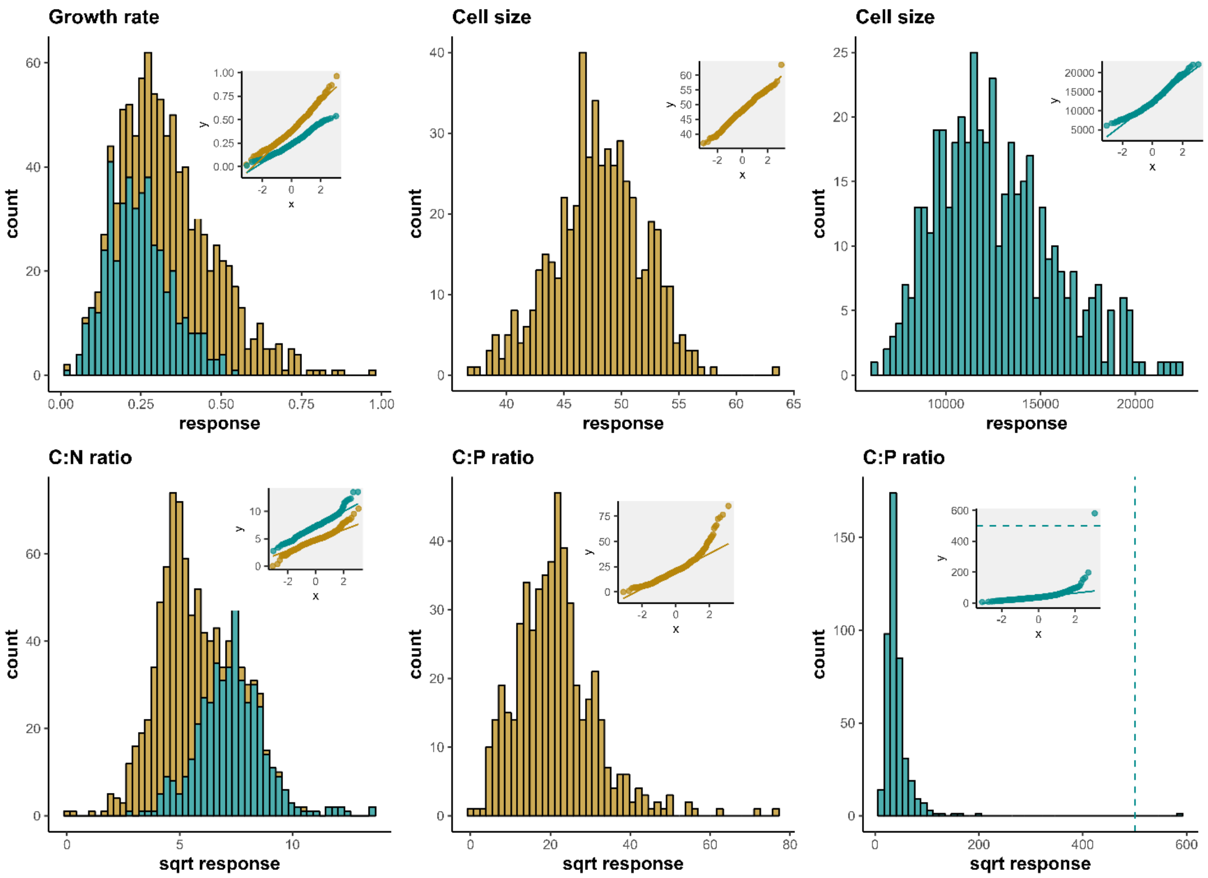

Figure S 4 Distribution of the response variables growth rate, cell size and stoichiometry used for the SEM. Colours represent the different species. Each histogram contains the *qqplot* of the two species. The dashed line in the C:P ratio plot for *Staurastrum* marks the range at which one outlier was excluded (the value above the dashed line was excluded). For some response variables, a sqrt-transformation for normal distribution was necessary (C:N and C:P ratio).

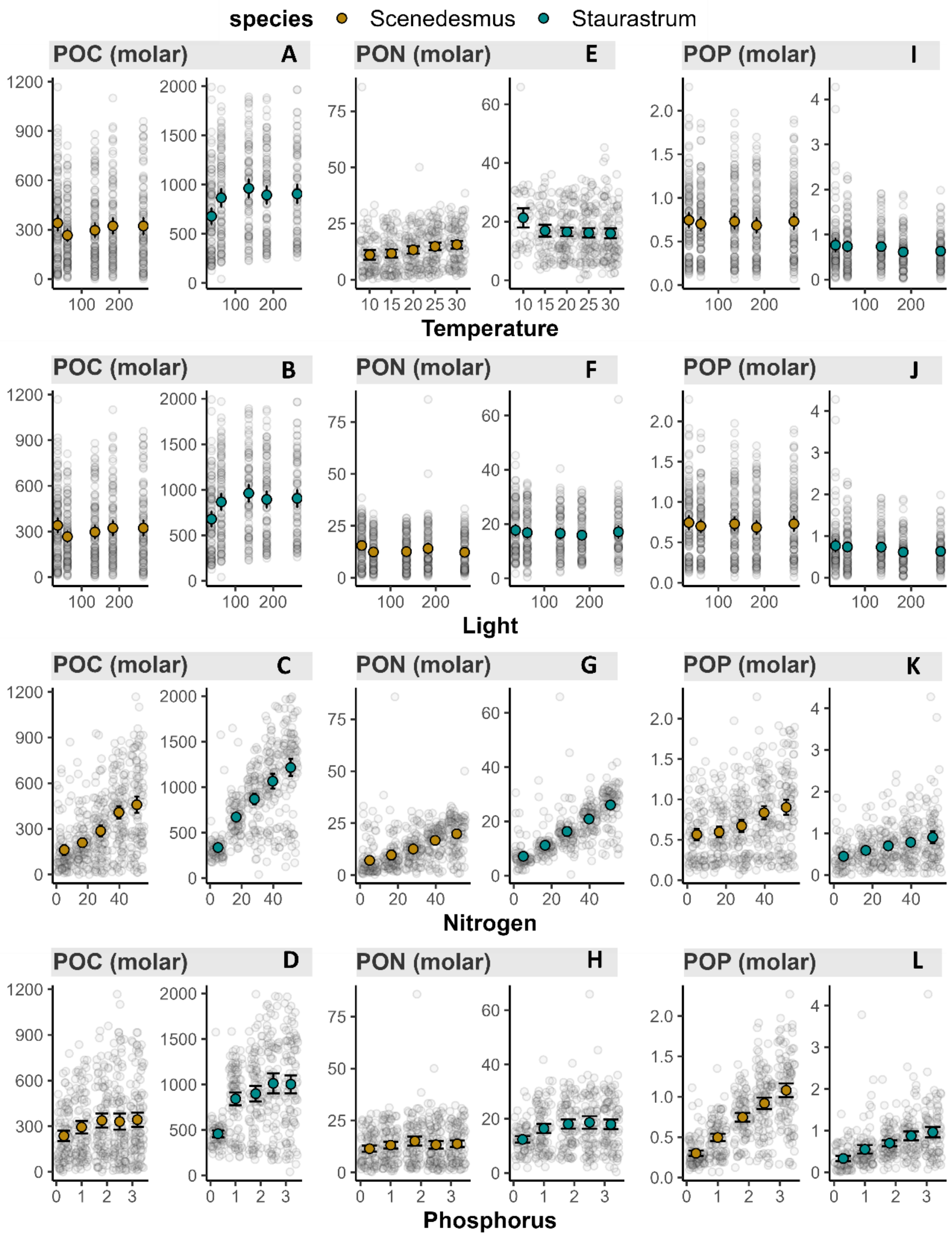

Figure S 5 Particulate organic Carbon (A-D), Nitrogen (E-H) and Phosphorus (I-L) along the gradients of increasing temperature, light intensity, nitrogen and phosphorus. The colored circles present the mean values and the error bars its standard errors for each treatment level. Grey circles present all observations.

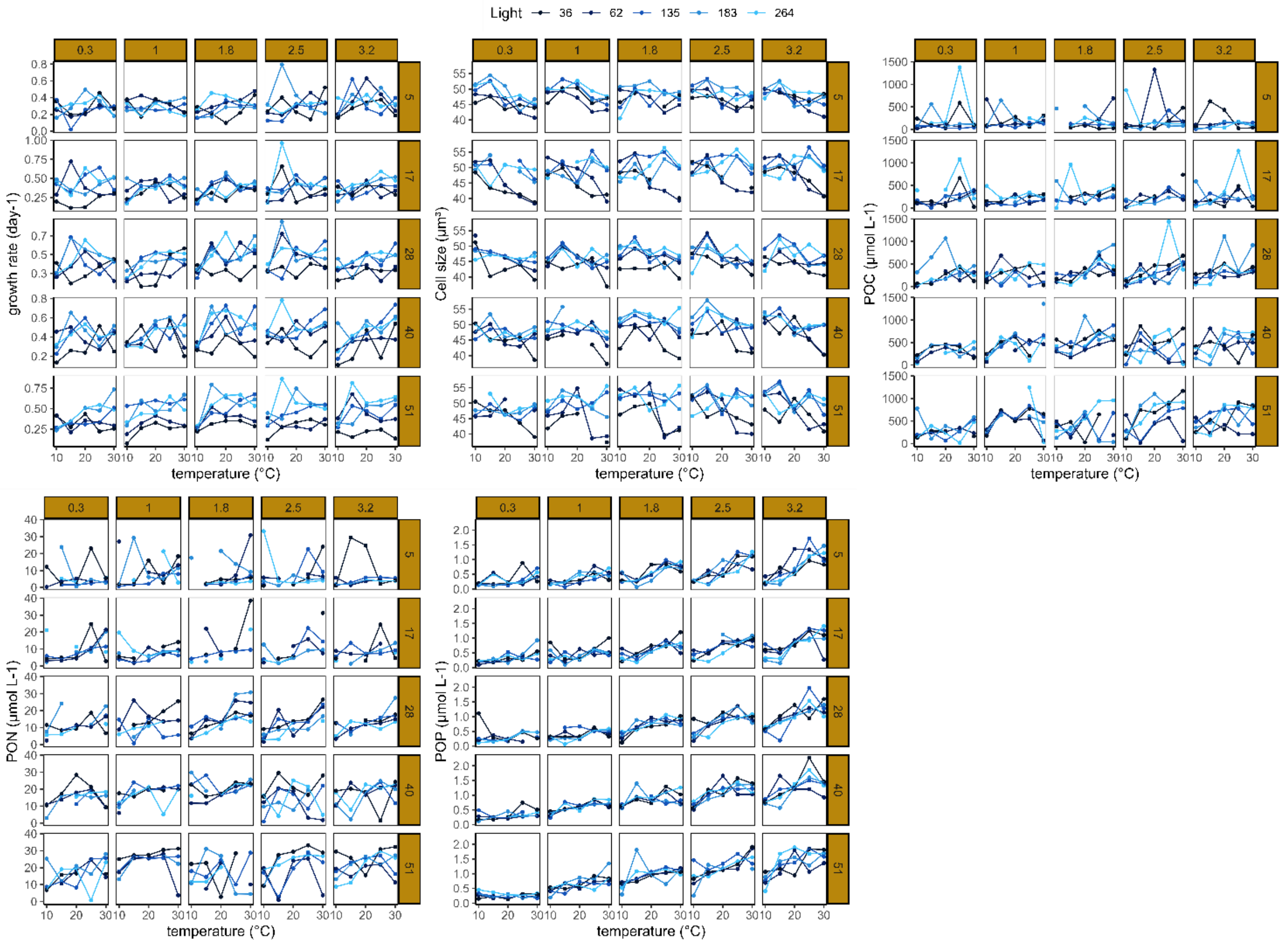

Figure S 6 Responses of *Scenedesmus* along the temperature gradient for each light intensity (blue colours) and nutrient concentrations (nitrogen: vertical panels, phosphorus: horizontal panels).

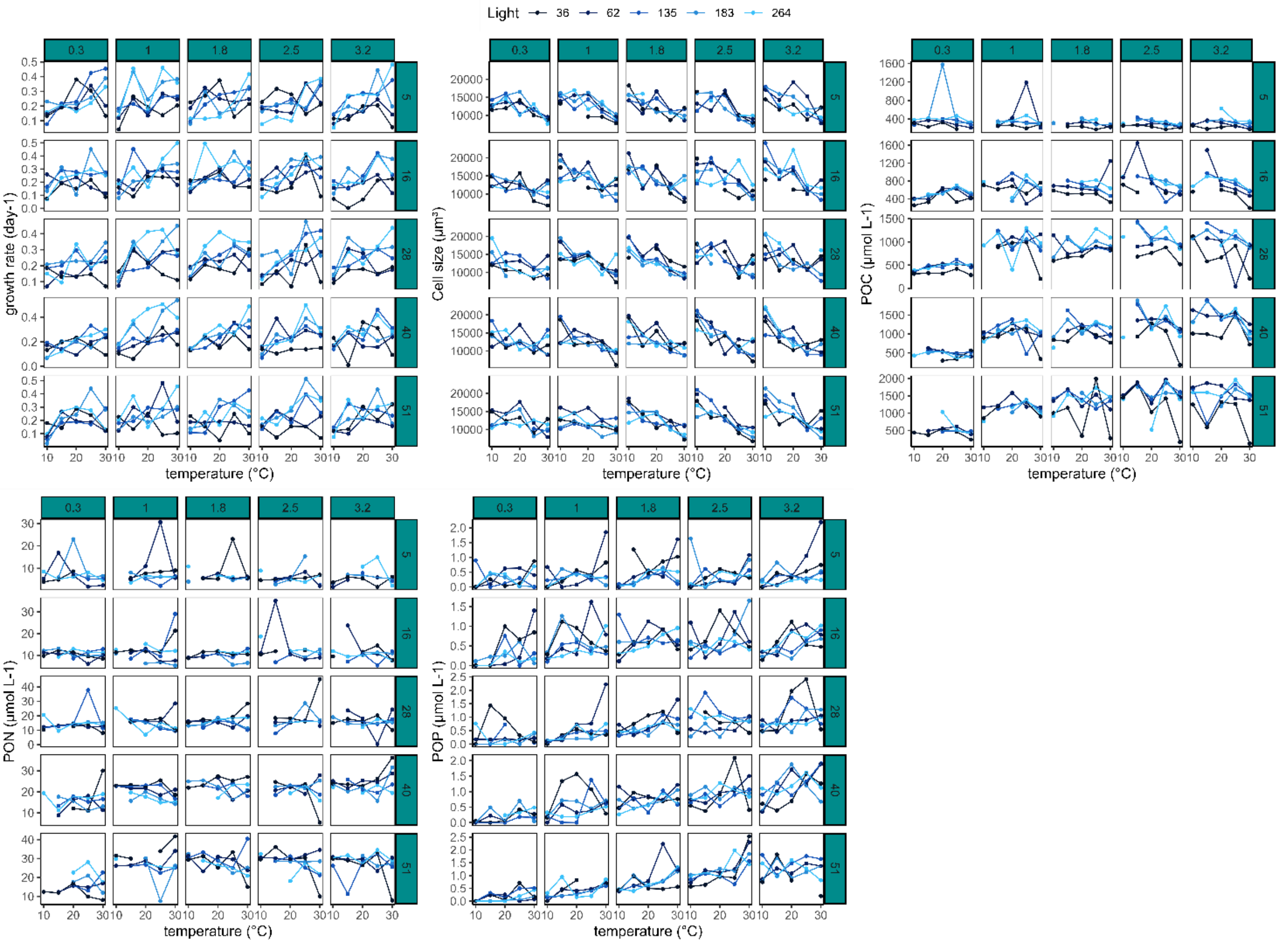

Figure S 7 Responses of *Staurastrum* along the temperature gradient for each light intensity (blue colours) and nutrient concentrations (nitrogen: vertical panels, phosphorus: horizontal panels).

Structural Equation Models for *Scenedesmus* and *Staurastrum*

For SEM, models containing only linear terms were compared with models containing non-linear terms using quadratic terms by AIC (see Figure S8-S11, and Table S1). For the responses in C:N ratio, no temperature effect was hypothesized. Therefore, models with and without a temperature term were compared (via AIC). Furthermore, we did not hypothesize a dependence of C:N ratio on growth. Therefore, models with and without C:N term were compared (via AIC). For *Scenedesmus*, the C:N effect on growth was excluded. The following models were used for the SEM, where * present terms were a quadratic term was added to the linear term. Note that C:N and C:P ratios were sqrt-transformed. In addition to the linear models, a correlation term between CN and cell size was added to the SEM.

$$growth_{scenedesmus} = temperature^{*} + light^{*} + N^{*} + P^{*} + cell\ size + CP \qquad \text{Eq. (1)}$$

$$growth_{staurastrum} = temperature^{*} + light^{*} + N^{*} + P^{*} + cell\ size + CP + CN \qquad \text{Eq. (2)}$$

$$cell\ size_{scenedesmus} = temperature^{*} + light^{*} + N^{*} + P^{*} \qquad \text{Eq. (3)}$$

$$cell\ size_{staurastrum} = temperature + light + N + P \qquad \text{Eq. (4)}$$

$$C{:}N_{scenedesmus} = temperature^{*} + light^{*} + N^{*} + P^{*} \qquad \text{Eq. (5)}$$

$$C{:}N_{staurastrum} = temperature^{*} + light^{*} + N^{*} + P^{*} \qquad \text{Eq. (6)}$$

$$C{:}P_{scenedesmus} = temperature + light + N + P \qquad \text{Eq. (7)}$$

$$C{:}P_{staurastrum} = temperature^{*} + light^{*} + N^{*} + P^{*} \qquad \text{Eq. (8)}$$

Table S 1 model comparison of models containing only linear terms and models containing non-linear terms. ΔAIC gives the AIC difference of the models containing non-linear terms to the linear model. Models with the lowest AIC was selected for the SEM.

		Growth ΔAIC	Cell size ΔAIC	C:N ratio ΔAIC	C:P ratio ΔAIC
Scenedesmus	Non-linear terms	-48	-58	- 6	+2
Staurastrum	Non-linear terms	- 18	+ 2	- 56	- 4

Table S 2 Standardized estimates of the non-linear terms in the used SEM. For the response in C:P ratio for *Scenedesmus*, only linear terms (i.e., linear effects) were included in the model due to a better AIC (see Table S1), and for *Staurastrum* the response in cell size only included linear effects due to a better AIC (see Table S1). Bold letters present significant estimates (p>0.05).

	Response	Std. Estimate of non-linear variables			
		temperature	light	N	P
Scenedesmus	growth	**-0.142**	**-0.126**	**-0.203**	-0.044
	cell size	**-0.125**	**-0.246**	**-0.118**	-0.059
	C:N ratio	**-0.149**	-0.004	0.045	-0.033
	C:P ratio	**-**	**-**	**-**	**-**
Staurastrum	growth	**-0.150**	**-0.089**	-0.018	**-0.115**
	cell size	**-**	**-**	**-**	**-**
	C:N ratio	**-0.166**	**-0.260**	**-0.136**	**-0.092**
	C:P ratio	-0.013	**-0.098**	-0.009	**0.115**

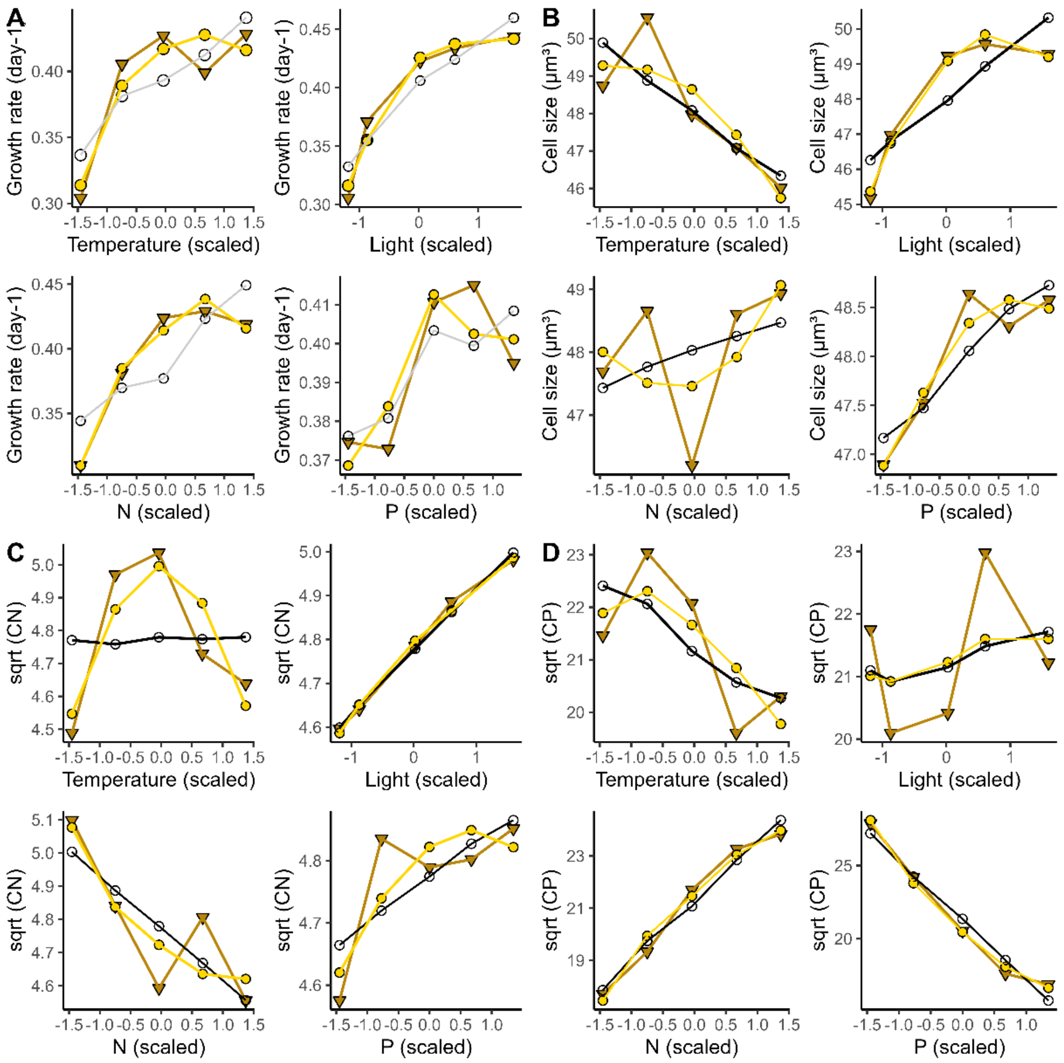

Figure S 8 Model validation for *Scenedesmus*. Comparison of responses predicted by linear models (black line) and linear models with non-linear terms (golden lines and circles) with observed responses (dark golden lines and triangles). Except for C:P ratio (D), non-linear terms were used for all responses. Note, C:N and C:P ratios are sqrt-transformed.

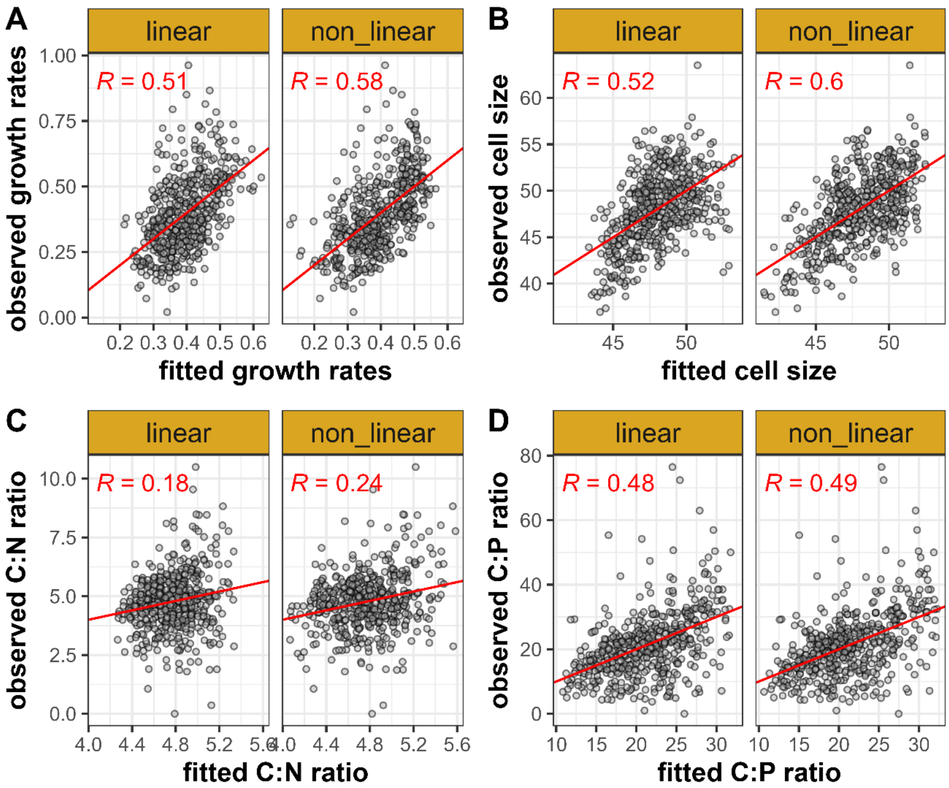

Figure S 9 Model validation for *Scenedesmus*. Comparison of responses predicted by linear models (linear) and linear models with non-linear terms (non_linear) with observed responses. Except for C:P ratio (D), non-linear terms were used for all responses. Red solid lines present regression line with the Pearson coefficient of correlation *R*. Note, C:N and C:P ratios are sqrt-transformed.

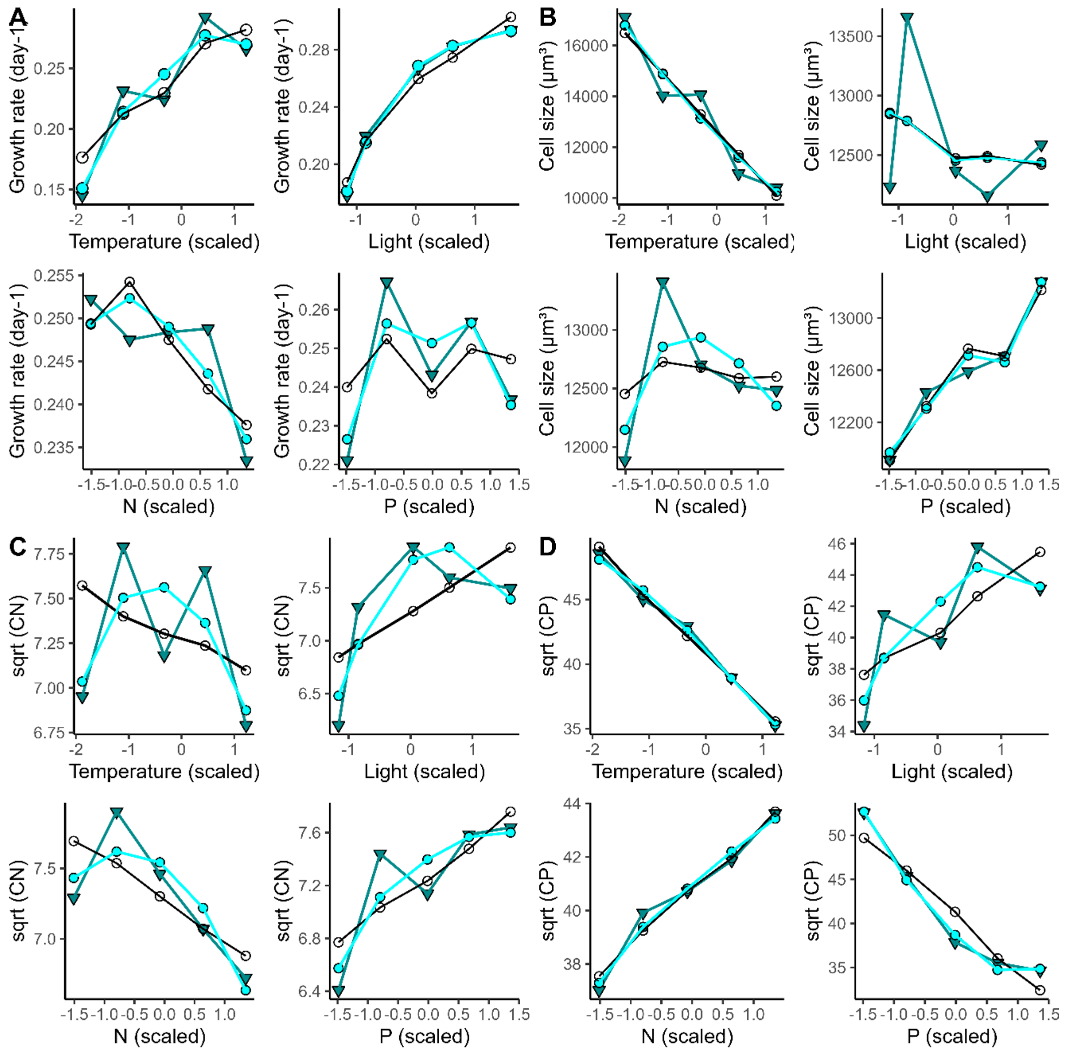

Figure S 10 Model validation for *Staurastrum*. Comparison of responses predicted by linear models (black line) and linear models with non-linear terms (turquoise) with observed responses (dark turquoise lines and triangles). Except for cell size (B) non-linear terms were used for all responses. Note, C:N and C:P ratios are sqrt-transformed.

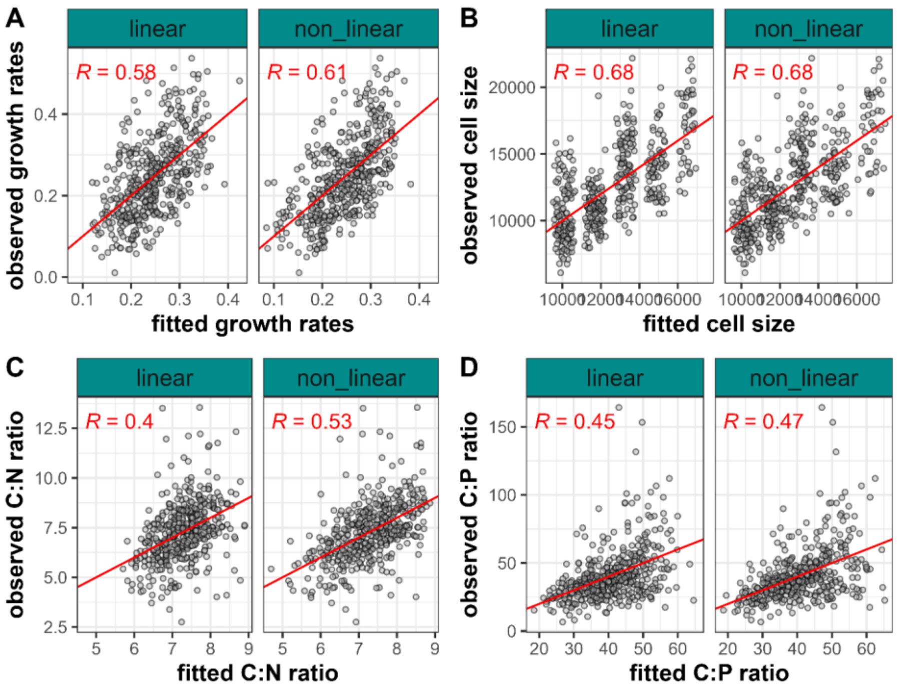

Figure S 11 Model validation for *Staurastrum*. Comparison of responses predicted by linear models (linear) and linear models with non-linear terms (non_linear) with observed responses. Except for cell size (B), non-linear terms were used for all responses. Red solid lines present regression lines with the Pearson coefficient of correlation *R*. Note, C:N and C:P ratios are sqrt-transformed.
